# Supplementary material for: Hot spot prediction in protein-protein interactions by an ensemble system
Source: BMC Syst Biol. 2018 Dec 31;12(Suppl 9):132. doi: 10.1186/s12918-018-0665-8 (PMC6311905; doi:10.1186/s12918-018-0665-8)
Supplement: Supplementary file 2 — All relevant MATLAB code. A simple Matlab implement of our predictor. (ZIP 19,742 kb) [file 12918_2018_665_MOESM2_ESM.zip › Additional file 2/Read ASA/ASA website/dbMPIKT/4BPXB.html]

 NetSurfP - Protein Surface Accessibility and Secondary Structure Predictions

  

|  |  |  |
| --- | --- | --- |
|  |  | NetSurfP - Protein Surface Accessibility and Secondary Structure Predictions  Technical University of Denmark |

  


---

```
# For publication of results, please cite:
# A generic method for assignment of reliability scores applied to solvent accessibility predictions.
# Bent Petersen, Thomas Nordahl Petersen, Pernille Andersen, Morten Nielsen and Claus Lundegaard
# BMC Structural Biology 2009, 9:51 doi:10.1186/1472-6807-9-51
#
# Column 1: Class assignment - B for buried or E for Exposed - Threshold: 25% exposure, but not based on RSA
# Column 2: Amino acid
# Column 3: Sequence name
# Column 4: Amino acid number
# Column 5: Relative Surface Accessibility - RSA
# Column 6: Absolute Surface Accessibility
# Column 7: Z-fit score for RSA prediction
# Column 8: Probability for Alpha-Helix
# Column 9: Probability for Beta-strand
# Column 10: Probability for Coil
E M  4BPXB                  1    0.756 151.316   0.045   0.003   0.003   0.994
E G  4BPXB                  2    0.614  48.361  -1.293   0.058   0.017   0.925
E Y  4BPXB                  3    0.506 108.111  -0.490   0.199   0.152   0.649
E S  4BPXB                  4    0.663  77.692   0.165   0.216   0.235   0.548
E E  4BPXB                  5    0.535  93.552   0.459   0.231   0.330   0.439
B V  4BPXB                  6    0.186  28.527   0.011   0.252   0.423   0.325
E N  4BPXB                  7    0.525  76.787  -0.350   0.252   0.423   0.325
E L  4BPXB                  8    0.356  65.275  -0.363   0.252   0.423   0.325
E S  4BPXB                  9    0.382  44.747  -0.397   0.231   0.330   0.439
E K  4BPXB                 10    0.411  84.563   0.704   0.148   0.418   0.435
B L  4BPXB                 11    0.172  31.402  -0.569   0.159   0.516   0.325
B F  4BPXB                 12    0.292  58.564  -0.710   0.169   0.612   0.219
E A  4BPXB                 13    0.465  51.298   0.247   0.159   0.516   0.325
E G  4BPXB                 14    0.407  32.031  -1.421   0.066   0.296   0.638
E C  4BPXB                 15    0.452  63.433  -0.532   0.064   0.216   0.721
E A  4BPXB                 16    0.597  65.745  -0.005   0.074   0.484   0.442
E V  4BPXB                 17    0.375  57.668   0.676   0.023   0.655   0.322
E K  4BPXB                 18    0.481  99.003   0.272   0.021   0.756   0.223
E S  4BPXB                 19    0.330  38.699  -0.754   0.021   0.756   0.223
E T  4BPXB                 20    0.485  67.339  -0.543   0.022   0.552   0.426
E G  4BPXB                 21    0.463  36.454  -1.215   0.022   0.359   0.619
E S  4BPXB                 22    0.406  47.630  -0.356   0.064   0.216   0.721
E T  4BPXB                 23    0.649  90.044  -0.308   0.052   0.084   0.864
E G  4BPXB                 24    0.510  40.161  -0.751   0.053   0.043   0.903
E S  4BPXB                 25    0.587  68.832  -0.427   0.056   0.142   0.802
E T  4BPXB                 26    0.535  74.163   0.218   0.066   0.296   0.638
E G  4BPXB                 27    0.306  24.082  -1.426   0.064   0.216   0.721
E S  4BPXB                 28    0.434  50.888  -0.371   0.064   0.216   0.721
E T  4BPXB                 29    0.569  78.906  -0.704   0.118   0.150   0.732
E G  4BPXB                 30    0.465  36.611  -0.420   0.052   0.084   0.864
E S  4BPXB                 31    0.414  48.521  -0.763   0.113   0.087   0.800
E T  4BPXB                 32    0.508  70.404  -1.144   0.052   0.084   0.864
E G  4BPXB                 33    0.349  27.451  -0.918   0.113   0.087   0.800
E S  4BPXB                 34    0.556  65.163  -0.852   0.113   0.087   0.800
E N  4BPXB                 35    0.538  78.719  -1.116   0.113   0.043   0.844
E A  4BPXB                 36    0.592  65.194  -1.380   0.113   0.043   0.844
E S  4BPXB                 37    0.503  58.998   0.047   0.113   0.043   0.844
B Y  4BPXB                 38    0.204  43.531   0.602   0.053   0.043   0.903
E P  4BPXB                 39    0.413  58.661  -0.656   0.053   0.043   0.903
E H  4BPXB                 40    0.335  60.900  -1.168   0.064   0.216   0.721
E C  4BPXB                 41    0.299  41.923   0.412   0.066   0.296   0.638
B L  4BPXB                 42    0.048   8.862   0.185   0.069   0.386   0.545
B Q  4BPXB                 43    0.229  40.899  -0.319   0.069   0.386   0.545
B F  4BPXB                 44    0.071  14.330  -0.179   0.069   0.386   0.545
B Y  4BPXB                 45    0.128  27.311  -0.051   0.069   0.386   0.545
E L  4BPXB                 46    0.516  94.498  -0.157   0.069   0.386   0.545
B Q  4BPXB                 47    0.226  40.292   1.089   0.056   0.142   0.802
B P  4BPXB                 48    0.273  38.753   0.360   0.018   0.019   0.964
B P  4BPXB                 49    0.216  30.679  -0.080   0.018   0.019   0.964
E S  4BPXB                 50    0.478  56.068  -0.379   0.018   0.019   0.964
E E  4BPXB                 51    0.494  86.302  -0.850   0.018   0.047   0.935
E N  4BPXB                 52    0.686 100.430   0.256   0.018   0.088   0.893
B I  4BPXB                 53    0.072  13.264   1.038   0.020   0.205   0.775
E S  4BPXB                 54    0.391  45.849   1.257   0.004   0.138   0.858
B L  4BPXB                 55    0.142  26.073   1.133   0.879   0.010   0.111
E I  4BPXB                 56    0.524  96.995   1.222   0.923   0.002   0.076
E E  4BPXB                 57    0.296  51.764   1.658   0.970   0.001   0.030
B F  4BPXB                 58    0.041   8.168   0.352   0.970   0.001   0.030
B E  4BPXB                 59    0.217  37.980   0.867   0.970   0.001   0.030
E N  4BPXB                 60    0.558  81.677   1.552   0.970   0.001   0.030
B L  4BPXB                 61    0.099  18.182   1.475   0.970   0.001   0.030
B A  4BPXB                 62    0.023   2.480   0.738   0.970   0.001   0.030
B I  4BPXB                 63    0.208  38.406   0.107   0.970   0.001   0.030
E D  4BPXB                 64    0.430  62.035   0.591   0.970   0.001   0.030
B R  4BPXB                 65    0.101  23.243   0.681   0.988   0.000   0.012
B V  4BPXB                 66    0.094  14.386   0.760   0.988   0.000   0.012
E K  4BPXB                 67    0.442  91.002   1.752   0.988   0.000   0.012
B L  4BPXB                 68    0.048   8.752   0.860   0.988   0.000   0.012
B L  4BPXB                 69    0.045   8.148   1.392   0.970   0.001   0.030
E K  4BPXB                 70    0.399  82.157   1.440   0.970   0.001   0.030
B S  4BPXB                 71    0.249  29.183   1.413   0.970   0.001   0.030
B V  4BPXB                 72    0.030   4.626   0.918   0.923   0.002   0.076
E E  4BPXB                 73    0.292  50.977   0.667   0.923   0.002   0.076
E N  4BPXB                 74    0.524  76.670   0.725   0.782   0.003   0.216
B L  4BPXB                 75    0.147  26.842   0.378   0.600   0.003   0.397
E G  4BPXB                 76    0.325  25.577  -0.497   0.502   0.002   0.495
E V  4BPXB                 77    0.473  72.777  -1.004   0.600   0.003   0.397
E S  4BPXB                 78    0.506  59.327  -0.865   0.406   0.004   0.590
E Y  4BPXB                 79    0.469 100.183  -0.667   0.176   0.004   0.820
E V  4BPXB                 80    0.397  61.019  -0.910   0.115   0.016   0.868
E K  4BPXB                 81    0.546 112.415  -0.066   0.115   0.016   0.868
E G  4BPXB                 82    0.467  36.714  -0.345   0.058   0.017   0.925
E T  4BPXB                 83    0.265  36.783  -0.561   0.622   0.015   0.363
E E  4BPXB                 84    0.654 114.201   0.187   0.858   0.002   0.139
E Q  4BPXB                 85    0.563 100.588   0.714   0.858   0.002   0.139
B Y  4BPXB                 86    0.135  28.764   0.040   0.923   0.002   0.076
E Q  4BPXB                 87    0.435  77.762   0.288   0.923   0.002   0.076
E S  4BPXB                 88    0.638  74.809   0.997   0.970   0.001   0.030
E K  4BPXB                 89    0.362  74.505   1.253   0.970   0.001   0.030
B L  4BPXB                 90    0.046   8.349  -0.182   0.970   0.001   0.030
E E  4BPXB                 91    0.396  69.181   1.385   0.970   0.001   0.030
E S  4BPXB                 92    0.617  72.277   1.261   0.970   0.001   0.030
E E  4BPXB                 93    0.277  48.374   0.917   0.923   0.002   0.076
B L  4BPXB                 94    0.078  14.337  -0.737   0.923   0.002   0.076
E R  4BPXB                 95    0.531 121.622   0.771   0.858   0.002   0.139
E K  4BPXB                 96    0.600 123.358  -0.080   0.782   0.003   0.216
B L  4BPXB                 97    0.263  48.119  -0.990   0.406   0.004   0.590
E K  4BPXB                 98    0.426  87.608  -1.843   0.181   0.016   0.803
E F  4BPXB                 99    0.336  67.435  -1.563   0.115   0.016   0.868
E S  4BPXB                100    0.459  53.842  -1.627   0.053   0.043   0.903
E Y  4BPXB                101    0.315  67.273  -1.178   0.052   0.084   0.864
E R  4BPXB                102    0.427  97.691  -0.954   0.018   0.047   0.935
E E  4BPXB                103    0.414  72.273  -2.292   0.018   0.019   0.964
E N  4BPXB                104    0.614  89.963  -0.913   0.018   0.019   0.964
B L  4BPXB                105    0.267  48.906  -0.756   0.016   0.005   0.979
E E  4BPXB                106    0.654 114.306  -0.585   0.176   0.004   0.820
E D  4BPXB                107    0.628  90.480  -0.727   0.502   0.002   0.495
E E  4BPXB                108    0.398  69.496  -0.569   0.694   0.003   0.303
E Y  4BPXB                109    0.321  68.705  -0.583   0.858   0.002   0.139
E E  4BPXB                110    0.495  86.424   0.187   0.923   0.002   0.076
E P  4BPXB                111    0.435  61.741  -0.297   0.923   0.002   0.076
E R  4BPXB                112    0.317  72.479   0.233   0.879   0.010   0.111
E R  4BPXB                113    0.306  70.166   0.012   0.802   0.014   0.185
E R  4BPXB                114    0.436  99.752   0.566   0.694   0.003   0.303
E D  4BPXB                115    0.361  52.078  -0.820   0.694   0.003   0.303
B H  4BPXB                116    0.115  20.955   0.723   0.802   0.014   0.185
B I  4BPXB                117    0.056  10.360   0.197   0.802   0.014   0.185
B S  4BPXB                118    0.087  10.232   0.236   0.802   0.014   0.185
B H  4BPXB                119    0.138  25.139   0.287   0.802   0.014   0.185
B F  4BPXB                120    0.076  15.233   0.921   0.879   0.010   0.111
B I  4BPXB                121    0.048   8.954   0.108   0.911   0.033   0.057
B L  4BPXB                122    0.039   7.178  -0.125   0.911   0.033   0.057
B R  4BPXB                123    0.140  32.083   0.157   0.911   0.033   0.057
B L  4BPXB                124    0.063  11.462  -0.121   0.911   0.033   0.057
B A  4BPXB                125    0.061   6.689  -0.146   0.831   0.044   0.125
B Y  4BPXB                126    0.166  35.496  -0.530   0.701   0.107   0.192
B C  4BPXB                127    0.101  14.124  -0.494   0.268   0.043   0.689
E Q  4BPXB                128    0.438  78.209  -0.159   0.053   0.043   0.903
E S  4BPXB                129    0.379  44.419  -0.001   0.058   0.017   0.925
E E  4BPXB                130    0.626 109.292   0.626   0.858   0.002   0.139
E E  4BPXB                131    0.630 109.991   0.628   0.923   0.002   0.076
B L  4BPXB                132    0.197  36.089   0.840   0.970   0.001   0.030
B R  4BPXB                133    0.147  33.732   0.708   0.970   0.001   0.030
E R  4BPXB                134    0.476 109.027   1.305   0.970   0.001   0.030
E W  4BPXB                135    0.282  67.749   1.334   0.970   0.001   0.030
B F  4BPXB                136    0.040   7.988  -0.235   0.970   0.001   0.030
B I  4BPXB                137    0.066  12.173   0.560   0.970   0.001   0.030
E Q  4BPXB                138    0.472  84.228   1.408   0.970   0.001   0.030
B Q  4BPXB                139    0.155  27.754   0.650   0.970   0.001   0.030
B E  4BPXB                140    0.048   8.316  -0.186   0.970   0.001   0.030
B M  4BPXB                141    0.095  19.070   0.502   0.970   0.001   0.030
E D  4BPXB                142    0.404  58.288   0.955   0.988   0.000   0.012
B L  4BPXB                143    0.052   9.466   0.569   0.988   0.000   0.012
B L  4BPXB                144    0.038   6.885   0.423   0.970   0.001   0.030
E R  4BPXB                145    0.274  62.860   1.689   0.970   0.001   0.030
B F  4BPXB                146    0.202  40.521   0.689   0.938   0.007   0.055
B R  4BPXB                147    0.186  42.548   0.714   0.879   0.010   0.111
B F  4BPXB                148    0.098  19.608   0.341   0.694   0.003   0.303
E S  4BPXB                149    0.591  69.265   0.968   0.406   0.004   0.590
E I  4BPXB                150    0.434  80.271  -0.344   0.176   0.004   0.820
E L  4BPXB                151    0.376  68.864  -0.247   0.053   0.005   0.942
E P  4BPXB                152    0.493  69.900   0.455   0.018   0.019   0.964
E K  4BPXB                153    0.569 117.105  -0.249   0.858   0.002   0.139
E D  4BPXB                154    0.694 100.020   0.803   0.923   0.002   0.076
E K  4BPXB                155    0.350  72.077   0.857   0.923   0.002   0.076
B I  4BPXB                156    0.108  19.924   0.762   0.970   0.001   0.030
E Q  4BPXB                157    0.489  87.318   1.241   0.970   0.001   0.030
E D  4BPXB                158    0.515  74.197   1.650   0.970   0.001   0.030
B F  4BPXB                159    0.078  15.574  -0.059   0.970   0.001   0.030
B L  4BPXB                160    0.111  20.269  -0.252   0.923   0.002   0.076
E K  4BPXB                161    0.627 129.056   0.761   0.923   0.002   0.076
E D  4BPXB                162    0.589  84.875   0.041   0.782   0.003   0.216
E S  4BPXB                163    0.352  41.219   0.352   0.246   0.004   0.750
E Q  4BPXB                164    0.725 129.556   0.003   0.016   0.005   0.979
B L  4BPXB                165    0.110  20.178   0.106   0.003   0.003   0.994
E Q  4BPXB                166    0.619 110.643   1.151   0.005   0.015   0.979
B F  4BPXB                167    0.160  32.152   0.361   0.004   0.138   0.858
E E  4BPXB                168    0.679 118.621   0.707   0.005   0.336   0.660
E A  4BPXB                169    0.498  54.902   0.657   0.005   0.336   0.660
B I  4BPXB                170    0.091  16.909   0.230   0.004   0.138   0.858
E S  4BPXB                171    0.518  60.698   0.689   0.018   0.019   0.964
E D  4BPXB                172    0.623  89.745   1.129   0.923   0.002   0.076
E E  4BPXB                173    0.687 119.949   1.110   0.923   0.002   0.076
B E  4BPXB                174    0.189  32.983  -0.209   0.923   0.002   0.076
B K  4BPXB                175    0.228  46.817   0.595   0.923   0.002   0.076
E T  4BPXB                176    0.569  78.851   0.589   0.858   0.002   0.139
E L  4BPXB                177    0.411  75.254   0.541   0.858   0.002   0.139
B R  4BPXB                178    0.163  37.373   0.716   0.782   0.003   0.216
E E  4BPXB                179    0.352  61.564   0.484   0.802   0.014   0.185
E Q  4BPXB                180    0.600 107.071   0.410   0.717   0.014   0.269
E E  4BPXB                181    0.424  74.020   0.218   0.622   0.015   0.363
B I  4BPXB                182    0.051   9.435   0.186   0.622   0.015   0.363
B V  4BPXB                183    0.263  40.392   0.317   0.561   0.047   0.393
E A  4BPXB                184    0.365  40.245  -0.550   0.561   0.047   0.393
E S  4BPXB                185    0.394  46.165  -0.396   0.354   0.048   0.598
B S  4BPXB                186    0.279  32.675  -1.072   0.184   0.043   0.773
E P  4BPXB                187    0.430  60.989  -1.546   0.113   0.043   0.844
E S  4BPXB                188    0.577  67.601  -0.536   0.052   0.084   0.864
B L  4BPXB                189    0.192  35.174  -0.130   0.118   0.150   0.732
E S  4BPXB                190    0.566  66.382   0.080   0.118   0.150   0.732
E G  4BPXB                191    0.507  39.869  -0.946   0.118   0.150   0.732
E L  4BPXB                192    0.459  84.025  -1.243   0.118   0.150   0.732
E K  4BPXB                193    0.598 123.070   0.220   0.118   0.150   0.732
E L  4BPXB                194    0.324  59.306  -0.535   0.118   0.150   0.732
E G  4BPXB                195    0.567  44.623   0.042   0.056   0.142   0.802
E F  4BPXB                196    0.546 109.582   0.091   0.019   0.141   0.840
E E  4BPXB                197    0.464  81.096   0.903   0.022   0.359   0.619
E S  4BPXB                198    0.389  45.614   1.102   0.004   0.616   0.381
B I  4BPXB                199    0.130  24.087   0.643   0.001   0.900   0.099
B Y  4BPXB                200    0.125  26.627   0.809   0.001   0.959   0.040
E K  4BPXB                201    0.373  76.747   0.703   0.001   0.900   0.099
B I  4BPXB                202    0.044   8.196   0.967   0.003   0.718   0.279
E P  4BPXB                203    0.349  49.481   1.025   0.005   0.336   0.660
B F  4BPXB                204    0.077  15.434   0.271   0.056   0.142   0.802
E A  4BPXB                205    0.506  55.794   0.390   0.199   0.152   0.649
E D  4BPXB                206    0.512  73.822   0.852   0.199   0.152   0.649
B A  4BPXB                207    0.053   5.885   0.947   0.354   0.048   0.598
B L  4BPXB                208    0.208  38.140   0.540   0.354   0.048   0.598
E D  4BPXB                209    0.568  81.878   0.705   0.354   0.048   0.598
B L  4BPXB                210    0.170  31.072   0.154   0.354   0.048   0.598
B F  4BPXB                211    0.106  21.174   0.347   0.386   0.097   0.517
E R  4BPXB                212    0.558 127.736   1.005   0.278   0.093   0.628
E G  4BPXB                213    0.549  43.238   0.724   0.118   0.150   0.732
B R  4BPXB                214    0.251  57.456   0.717   0.021   0.451   0.528
E K  4BPXB                215    0.280  57.678   1.229   0.018   0.846   0.136
B V  4BPXB                216    0.044   6.778   0.776   0.011   0.918   0.071
B Y  4BPXB                217    0.139  29.704   1.745   0.006   0.962   0.032
B L  4BPXB                218    0.043   7.855   0.689   0.011   0.918   0.071
E E  4BPXB                219    0.344  60.149   0.569   0.021   0.756   0.223
E D  4BPXB                220    0.543  78.217   0.776   0.019   0.141   0.840
B G  4BPXB                221    0.143  11.238  -0.941   0.004   0.138   0.858
E F  4BPXB                222    0.275  55.233   0.494   0.021   0.756   0.223
B A  4BPXB                223    0.031   3.372  -0.517   0.001   0.900   0.099
B Y  4BPXB                224    0.224  47.762   1.106   0.001   0.900   0.099
B V  4BPXB                225    0.035   5.426   1.037   0.003   0.718   0.279
B P  4BPXB                226    0.155  21.994   1.149   0.005   0.262   0.733
E L  4BPXB                227    0.334  61.137   0.689   0.430   0.016   0.555
E K  4BPXB                228    0.611 125.621   0.709   0.694   0.003   0.303
B D  4BPXB                229    0.253  36.529   0.293   0.782   0.003   0.216
B I  4BPXB                230    0.056  10.397   0.315   0.879   0.010   0.111
B V  4BPXB                231    0.244  37.549   0.771   0.938   0.007   0.055
E A  4BPXB                232    0.450  49.623   0.710   0.975   0.003   0.022
B I  4BPXB                233    0.086  15.966   1.280   0.970   0.001   0.030
B I  4BPXB                234    0.043   7.863   0.896   0.988   0.000   0.012
B L  4BPXB                235    0.141  25.744   0.645   0.988   0.000   0.012
E N  4BPXB                236    0.382  55.954   0.956   0.988   0.000   0.012
E E  4BPXB                237    0.334  58.402   1.892   0.988   0.000   0.012
B F  4BPXB                238    0.055  11.099   0.454   0.970   0.001   0.030
E R  4BPXB                239    0.272  62.334   1.340   0.988   0.000   0.012
E A  4BPXB                240    0.564  62.120   1.109   0.970   0.001   0.030
E K  4BPXB                241    0.309  63.479   2.202   0.970   0.001   0.030
B L  4BPXB                242    0.031   5.676   0.590   0.970   0.001   0.030
E S  4BPXB                243    0.308  36.133   1.358   0.970   0.001   0.030
E K  4BPXB                244    0.538 110.769   1.372   0.970   0.001   0.030
B A  4BPXB                245    0.205  22.635   0.682   0.970   0.001   0.030
B L  4BPXB                246    0.056  10.290   0.626   0.970   0.001   0.030
E A  4BPXB                247    0.434  47.849   1.791   0.970   0.001   0.030
E L  4BPXB                248    0.400  73.185   0.642   0.970   0.001   0.030
B T  4BPXB                249    0.125  17.337   0.423   0.970   0.001   0.030
B A  4BPXB                250    0.252  27.781  -0.890   0.923   0.002   0.076
E R  4BPXB                251    0.552 126.408   0.543   0.858   0.002   0.139
E S  4BPXB                252    0.330  38.723  -0.285   0.782   0.003   0.216
B L  4BPXB                253    0.115  21.130   0.656   0.502   0.002   0.495
E P  4BPXB                254    0.448  63.600  -0.441   0.321   0.003   0.675
E A  4BPXB                255    0.507  55.838   0.295   0.246   0.004   0.750
B V  4BPXB                256    0.200  30.801  -0.138   0.181   0.016   0.803
E Q  4BPXB                257    0.470  83.978  -0.294   0.058   0.017   0.925
E S  4BPXB                258    0.633  74.188  -0.451   0.115   0.016   0.868
E D  4BPXB                259    0.372  53.576  -0.200   0.181   0.016   0.803
E E  4BPXB                260    0.618 107.982  -0.193   0.430   0.016   0.555
B R  4BPXB                261    0.254  58.166   0.348   0.522   0.016   0.462
B L  4BPXB                262    0.070  12.762   0.134   0.717   0.014   0.269
E Q  4BPXB                263    0.301  53.741   1.119   0.694   0.003   0.303
E P  4BPXB                264    0.449  63.727   0.282   0.782   0.003   0.216
B L  4BPXB                265    0.153  27.978   0.140   0.782   0.003   0.216
B L  4BPXB                266    0.168  30.834  -0.459   0.782   0.003   0.216
E N  4BPXB                267    0.695 101.792   1.000   0.694   0.003   0.303
E H  4BPXB                268    0.553 100.554   0.172   0.502   0.002   0.495
E L  4BPXB                269    0.492  90.067  -1.210   0.053   0.005   0.942

Explain the output.  Go back.
```

---
